# Fetal movement trials: Where is the evidence in settings with a high burden of stillbirths?

**DOI:** 10.1111/1471-0528.17249

**Published:** 2022-06-29

**Authors:** Natasha Housseine, Joyce Browne, Nanna Maaløe, Brenda Sequeira Dmello, Sam Ali, Muzdalifat Abeid, Tarek Meguid, Marcus J. Rijken, Hussein Kidanto

**Affiliations:** ^1^ Medical College East Africa Aga Khan University Dar es Salaam Tanzania; ^2^ Julius Centre for Health Sciences and Primary Care Julius Global Health University Medical Centre Utrecht, Utrecht University Utrecht the Netherlands; ^3^ Global Health Section Department of Public Health University of Copenhagen Copenhagen Denmark; ^4^ Comprehensive Community Based Rehabilitation in Tanzania Dar es salaam Tanzania; ^5^ Research Department Ernest Cook Ultrasound Research and Education Institute (ECUREI) Kampala Uganda; ^6^ Child Health Unit, Department of Paediatrics and Child Health University of Cape Town Cape Town South Africa; ^7^ Vrouw en Baby Department University Medical Centre Utrecht, Utrecht University Utrecht the Netherlands; ^8^ Obstetric Department Amsterdam University Medical Center Amsterdam the Netherlands

Fetal movement (FM) is a sign of fetal life and wellbeing that is felt by the pregnant woman, and reduced FM is known to precede stillbirths.^1^, ^2^ Therefore, healthcare providers may advise women to monitor and report if their babies’ movements are fewer than usual. In high‐income countries (HICs), there has been a renewed interest in FM with a recent wave of large‐scale randomised controlled clinical trials investigating its potential to reduce stillbirths. The My Baby’s Movement trial in Australia and New Zealand and the Mindfetalness trial in Sweden have investigated the effects of intervention aimed at increasing women’s awareness of FM.^3^, ^4^ In the UK, the AFFIRM trial investigated the effects of an FM awareness package coupled with a standardised management protocol.^5^ The ongoing CEPRA study in the Netherlands, UK and Australia aims to evaluate Cerebro Placental Ratio as an indicator for delivery in women with reduced FM.^6^ None of the completed trials, however, found significant reductions in stillbirths. Moreover, they showed conflicting results on some potential harmful consequences, such as increased rates of obstetric interventions. In this commentary, we reflect on these trials through a global lens, and we urgently call for more trials – but this time in settings suffering the majority (98%) of the world’s 2 million annual stillbirths.

Importantly, the global applicability of these HIC trials is questionable. They were conducted in settings where women are aware of the importance of reduced FM and are empowered to access the highest standards of care. The contextual realities of pregnancy care are vastly different in low‐ and middle‐income countries (LMICs), where antenatal care and health education are substandard. Women lack health information to self‐monitor and report reduced FM. Furthermore, antenatal clinics are often overcrowded and understaffed, and lack supplies, clinical guidelines and the adequate training of health workers. Recent estimates show stillbirth rates of as high as 22 per 1000 total births in sub‐Saharan Africa, compared with fewer than 3 per 1000 total births in HICs.^7^ Given the downward trend of stillbirths reported in all the HIC trials, it is possible that the completed trials may be demonstrating a lack of *evidence* rather than a lack of *effectiveness*. We hypothesise that involving women in their care, through training on how to monitor their baby’s movement, and when and how to respond, coupled with strengthening healthcare workers’ respect and response to women’s concerns on reduced FM, is a low‐cost intervention with potential to significantly reduce stillbirths in high‐burden LMICs.

Surprisingly, high‐quality studies from LMICs that have assessed the effect of FM interventions on perinatal deaths are lacking.^2^ Of note, the authors of the above‐mentioned trials did not consider the well‐known major differences in clinical context globally as a limitation while discussing the generalisability of their findings. In fact, the latest My Baby’s Movement trial was not even published with open access, thereby limiting access to less privileged clinicians, researchers and policymakers.^4^ This lack of a global perspective on the international health crisis of preventable stillbirths is an epistemic injustice and a missed opportunity.^8^ We are concerned that the results of the above trials could prematurely prompt policies discouraging the use of FM awareness among pregnant women.^9^ It is thus crucial that the lack of generic applicability of the findings of these trials is stressed, and that their high‐resource contexts are considered when developing global clinical guidelines and future research priorities. Notably, it has been seen too often how the unbalanced evidence produced from studies in HICs has had unintended harmful influences on clinical practice in LMICs.^10^ For instance, it appears that the breech trials from HICs have also led to policy change in LMICs, with an increased use of caesarean section in the case of breech presentation. However, the risk ratios of vaginal breech births versus caesarean sections differ dramatically between high‐resource and low‐resource settings, with lower surgical safety in LMICs.^11^, ^12^

The prevailing constraints in LMICs should stimulate innovation and creativity to design low‐cost solutions that strengthen three areas: (i) FM awareness and monitoring; (ii) diagnosis to identify babies truly at risk; and (3) care provision protocols of pregnant women with reduced FM to improve perinatal outcomes. Although such strategies or their evidence base are often lacking in LMICs, there is some evidence about possible low‐cost diagnostic approaches to assess fetal risk following reduced FM: for example, measuring maternal blood pressure, fetal heart rate and fundal height,^13^ or antenatal (handheld) ultrasound to detect and monitor high‐risk pregnancies. Measuring fetal blood flow in Doppler ultrasound studies has also been useful, particularly in detecting growth restriction.^6^, ^14^ Involving women and health workers in studies will ensure the consideration of health‐system constraints and allow these to be embedded in the design, implementation and evaluation of any new intervention. If proven effective, this will increase the chance of the seamless integration of the intervention into existing care, and positive perceptions by providers and pregnant women, and will not increase the burden on already overwhelmed healthcare workers.

Unfortunately, maternal perception of FM is still too often the *only* signal of complications in the absence of regular high‐quality antenatal checks,^15^ and there are possibly many babies’ lives lost by ignoring this danger sign. Given the burden of need and the context‐specific realities that determine the effectiveness of interventions, we hope that these recent waves of FM trials will continue into LMICs to investigate whether and how FM awareness coupled with a context‐tailored management protocol can reduce stillbirths.

## AUTHOR CONTRIBUTIONS

NH conceived and wrote the first draft. JB, NM, BSD and MJR contributed to subsequent drafting of the commentary. All authors revised the commentary for important intellectual content and approved the final version to be published and agree to be accountable for all aspects of the work.

## CONFLICT OF INTERESTS

None declared. Completed disclosure of interests form available to view online as supporting information.

## ETHICAL APPROVAL

No ethics approval applicable for this commentary.

## Supporting information


Appendix S1

Appendix S2

Appendix S3

Appendix S4

Appendix S5

Appendix S6

Appendix S7

Appendix S8

Appendix S9

## Data Availability

Data sharing not applicable ‐ no new data generated, or the article describes entirely theoretical research.
